# Increased serum anti-CYP2E1 IgG autoantibody levels may be involved in the pathogenesis of occupational trichloroethylene hypersensitivity syndrome: a case–control study

**DOI:** 10.1007/s00204-022-03326-x

**Published:** 2022-06-28

**Authors:** Tamie Nakajima, Hailan Wang, Yuan Yuan, Yuki Ito, Hisao Naito, Yoshiyuki Kawamoto, Kozue Takeda, Kiyoshi Sakai, Na Zhao, Hongling Li, Xinxiang Qiu, Lihua Xia, Jiabin Chen, Qifeng Wu, Laiyu Li, Hanlin Huang, Yukie Yanagiba, Hiroshi Yatsuya, Michihiro Kamijima

**Affiliations:** 1 Research Institute of Life and Health Sciences, Chubu University, Kasugai, Aichi 487-8501 Japan; 2 Laboratory of Key Technology Research, Guangdong Province Hospital for Occupational Disease Prevention and Treatment, Guangzhou, Guangdong People’s Republic of China; 3 Department of Occupational and Environmental Health, Nagoya City University Graduate School of Medical Sciences, Nagoya, Aichi Japan; 4 College of Human Life and Environment, Kinjo Gakuin University, Nagoya, Aichi Japan; 5 College of Life and Health Sciences, Chubu University, Kasugai, Aichi Japan; 6 Poison Control Center Guangdong Province Hospital for Occupational Disease Prevention and Treatment, Guangzhou, Guangdong People’s Republic of China; 7 Guangdong Province Hospital for Women and Children Healthcare, Guangdong, People’s Republic of China; 8 Division of Industrial Toxicology and Biological Monitoring, National Institute of Occupational Safety and Health, Kawasaki, Kanagawa Japan; 9 Department of Public Health and Health Systems, Nagoya University Graduate School of Medicine, Nagoya, Aichi Japan

**Keywords:** Alanine aminotransferase, Case–control study, Anti-cytochrome P450 2E1 autoantibody (IgG), HLA-B*13:01, Hypersensitivity syndrome, Trichloroethylene

## Abstract

Occupational exposure to trichloroethylene (TCE) causes a systemic skin disorder with hepatitis known as TCE hypersensitivity syndrome (TCE-HS). Human Leukocyte Antigen (HLA)-B*13:01 is its susceptibility factor; however, the immunological pathogenesis of TCE-HS remains unknown. We herein examined the hypothesis that autoantibodies to CYP2E1 are primarily involved in TCE-HS. A case–control study of 80 TCE-HS patients, 186 TCE-tolerant controls (TCE-TC), and 71 TCE-nonexposed controls (TCE-nonEC) was conducted to measure their serum anti-CYP2E1 antibody (IgG) levels. The effects of TCE exposure indices, such as 8-h time-weighted-average (TWA) airborne concentrations, urinary metabolite concentrations, and TCE usage duration; sex; smoking and drinking habits; and alanine aminotransferase (ALT) levels on the antibody levels were also analyzed in the two control groups. There were significant differences in anti-CYP2E1 antibody levels among the three groups: TCE-TC > TCE-HS patients > TCE-nonEC. Antibody levels were not different between HLA-B*13:01 carriers and noncarriers in TCE-HS patients and TCE-TC. The serum CYP2E1 measurement suggested increased immunocomplex levels only in patients with TCE-HS. Multiple regression analysis for the two control groups showed that the antibody levels were significantly higher by the TCE exposure. Women had higher antibody levels than men; however, smoking, drinking, and ALT levels did not affect the anti-CYP2E1 antibody levels. Anti-CYP2E1 antibodies were elevated at concentrations lower than the TWA concentration of 2.5 ppm for TCE exposure. Since HLA-B*13:01 polymorphism was not involved in the autoantibody levels, the possible mechanism underlying the pathogenesis of TCE-HS is that TCE exposure induces anti-CYP2E1 autoantibody production, and HLA-B*13:01 is involved in the development of TCE-HS.

**Supplementary Information:**

The online version contains supplementary material available at 10.1007/s00204-022-03326-x.

## Introduction

Trichloroethylene (TCE), CCl_2_CHCl, (CAS No. 79-01-6) is still used worldwide as a solvent for removing oils, greases, waxes, tars, and moisture before final surface treatments such as galvanizing, electroplating, painting, anodizing, and application of conversion coatings (IARC Working Group on the Evaluation of Carcinogenic Risks to Humans 2014). Of numerous TCE-induced occupational diseases, TCE hypersensitivity syndrome (TCE-HS) has been focused on during the last 20 years (Huang et al. 2002, 2006; Kamijima et al. 2007; Wang et al. 2020). This disease is characterized by clinical manifestations that all the patients had systemic skin rash, hepatitis, fever, leukocytosis, lymphadenopathy, and human herpesvirus 6 (HHV6) reactivation (Huang et al. 2006; Kamijima et al. 2013; Watanabe et al. 2010) after commencement of TCE exposure about 1 month. The precise disease prevalence is unknown but was reportedly in the range of 0.25–12.5% in the involved workshops. The geographical characteristic is that this disease has often been reported in the southern part of China and Southeast Asian countries, but rarely in Europe, America, and Japan (Kamijima et al. 2007). This observation may be explained by the frequency of Human Leukocyte Antigen (HLA)-B*13:01 polymorphism, a susceptibility gene for TCE-HS development (Dai et al. 2020; Li et al. 2007; Wang et al. 2020). However, it is unclear how this gene acts in developing HS. It is also interesting from an industrial hygiene standpoint that, as mentioned earlier, the disease is not caused by a one-time exposure to TCE but rather is caused by approximately 1-month repeated exposures. TCE may not induce acute immune effects, but some immunotoxic substances, which have not been clarified yet, may be accumulated during the repeated TCE exposure.

CYP2E1, an isozyme of the drug-metabolizing enzyme cytochrome P450 (CYP), mostly oxidizes volatile hydrocarbons such as TCE (Nakajima 1997) and can be an antigen to produce anti-CYP2E1 autoantibodies that cause hepatitis (Sutti et al. 2014). The most well-known example is “halothane hepatitis.” Halothane (CHClBrClF_3_), an anesthetic ethane, is metabolized by CYP2E1 to produce trifluoroacetic adducts, and the repeated exposure produced CYP2E1 autoantibodies (autoimmune antibodies), which are believed to be the major cause of hepatitis (Eliasson and Kenna 1996; Kenna and Neuberger 1995). 1,1-Dichloro-2,2,2-trifluoroethane (CHCl_2_CF_3_, HCFC-123), used as an alternative to chlorofluorocarbons, is also metabolized by CYP2E1 to produce the same metabolites as halothane (White and De Matteis 2001). In 1997, Hoet et al. (1997) reported that anti-CYP2E1 autoantibodies, the same as those found in halothane hepatitis, were detected in biopsy liver specimens and sera in refrigerant workers who used HCFC-123.

Thus, the structural similarity of TCE to these two halogenated ethanes and animal studies revealing the involvement of CYP2E1 in TCE-induced acute liver injury (Nakajima et al. 1988; Ramdhan et al. 2008) led to the hypothesis that anti-CYP2E1 autoantibody production might be induced in patients with TCE-HS, thereby causing the associated hepatotoxicity.

In the present study, we tested this hypothesis using sera collected from TCE-HS patients, TCE-tolerant controls (TCE-TC), and TCE-nonexposed controls (TCE-nonEC).

## Materials and methods

### Study population

This study was conducted with the approval of the ethics committees of Nagoya City University (approval number 60-00-0505), Chubu University (approval number 20200085), and the Guangdong Province Hospital for Occupational Disease Prevention and Treatment (GPHODPT) (IRB#2,013,009; IRB#2,013,010) and was performed in accordance with the ethical standards of the 1964 Declaration of Helsinki and its later amendments. All subjects were Han Chinese, and all subjects provided written informed consent prior to their inclusion in the study.

The TCE-HS group consisted of 80 people diagnosed with TCE-HS between 2004 and 2009 and admitted to the GPHODPT. TCE-HS diagnosis was made according to the Chinese National Diagnostic Criteria of Occupational Medicamentosa-like Dermatitis due to TCE (GBZ 185–2006) (Ministry of Health of the People’s Republic of China 2007). The disease developed within an average of 27 days after TCE exposure (Huang et al. 2006; Kamijima et al. 2013). Many of the patients had been transferred from other hospitals to receive more advanced medical care and/or to obtain workers’ compensation. Therefore, blood samples were collected from 2 to 46 days after TCE exposure, although the sampling was generally made on the first day of hospitalization. In addition, serum biochemical data such as alanine aminotransferase (ALT) levels were also measured at the time of blood collection on admission. At the same time, alcohol consumption and smoking history were asked.

To know the TCE exposure status of the patients, we investigated on-site TCE exposure concentrations along with the liver function of 186 TCE-TC, i.e., all the workers who did not suffer from TCE-HS in 6 factories where TCE-HS patients occurred and other 6 factories without the disease occurrence between 2002 and 2010 were invited to participate in this study. Their individual exposure was measured with diffusive samplers, and the blood and urine were collected immediately after their shifts. Urine and serum were stored at − 30 °C and − 80 °C until use, respectively. In addition, a questionnaire was used to investigate the number of days after the commencement of TCE use and smoking and drinking histories.

To understand the effect of TCE on anti-CYP2E1 autoantibody induction, we recruited 71 TCE-nonEC whose sex and age matched those of TCE-HS patients: 31 from 450 thermal power plant workers who visited the GPHODPT to undergo health checkups in March 2012, and 35 and 5 from outpatient visits in March 2016 and 2017, respectively. Blood samples were taken, and serum ALT was measured, and the residual serum was stored at − 80 °C until the measurement of anti-CYP2E1 antibody levels. In addition, as with the TCE-TC, smoking and drinking histories were investigated with a questionnaire.

### Measurement of ALT

Serum ALT levels were measured using a dry, clinical chemistry analyzer Spotchem D-concept (Arkray, Kyoto, Japan). The normal range of ALT levels for this instrument was less than 10–42 IU/L for men and less than 10–23 IU/L for women. 10/√2 = 7 was used for the values of subjects with less than 10. The values measured among the institutes were confirmed to be similar.

### HLA-B*13:01 gene polymorphism

The HLA-B*13:01 polymorphism in TCE-HS patients and TCE-TC was measured as described previously (Wang et al. 2020), in which a part of data was already reported.

### Time-weighted average (TWA) of TCE exposure concentrations for TCE-tolerant controls

We determined the TCE exposure status of 159 out of 186 TCE-TC. Workers wore an organic gas monitor (SIBATA SCIENTIFIC TECHNOLOGY, Soka, Japan) on the collar of their work clothes near their breathing area just before work session, and the monitor was collected after their shifts. It was stored in a refrigerator at 4 °C until measurements. Adsorbed organic solvents were desorbed with carbon disulfide according to the company’s instructions, and TCE concentration was measured by gas chromatography-mass spectrometry (GC–MS) (5890 Series II/5971A, Agilent Technologies, Santa Clara, CA, USA). The limit of detection (LOD) concentration was less than 0.1 mg/m^3^ (Kamijima et al. 2008).

### Urinary metabolite concentrations in TCE-tolerant controls

Urinary TCE metabolites trichloroacetic acid (TCA) and trichloroethanol (TCEOH) in TCE-TC were measured using a method reported by Ramdhan et al. (2008) with a slight modification; n-hexane–ethyl acetate was used instead of n-hexane-dichloromethane as the extraction solvent for chromatographic separation. Briefly, β-glucuronidase (196 unit) was added to a 200-μL urine sample, followed by incubation at 37 °C for 16 h. Five microliters of dichloroacetic acid was added to the sample as an internal standard. The solution was then adjusted to approximately pH 3 with 0.1 M sulfuric acid and derivatized by adding 500 μL of water – 0.1 M sulfuric acid–methanol (6:5:1). The mixture was heated and shaken in a thermostatic bath at 70 °C for 10 min. After cooling to room temperature, 500 µL of n-hexane–ethyl acetate (1:1) was added to the mixture, and the mixture was then shaken for 20 min. After leaving the organic solution for 2 min, the vial was centrifuged at 900 g for 15 min. Finally, the supernatant solution (1 μL) was injected into GC–MS (6890 N, 5975; Agilent Technologies). The detection limits of TCA and TCEOH were 0.10 mg/l and 0.022 mg/l, respectively, which were the same as previously reported.

### Production of CYP2E1 protein

CYP2E1 protein was synthesized as follows. RNA was extracted from HepG2 cells using RNeasy Plus Mini kit (QIAGEN, Tokyo, Japan) and cDNA was synthesized by c-DNA-Synthesis kit (Thermo Fisher Scientific K.K., Tokyo, Japan). This was used as a template, and the CYP2E1 gene was amplified with primers 5′-ATGTCTGCCCTCGGAGTCACCGT-3′ and 5′-TGAAGCGGGAATGACACAGTTTGTAACG-3′ using LA-Taq. The PCR products were recombined into pcDNA^™^ 3.1/V5-His TOPO^®^ TA vector and cloned. The purified recombinant vectors were introduced into NIH3T3 cells and cultured. RIPA buffer was added to the cultured cells, and human CYP2E1 was extracted from the cultured cells.

The extracts were mixed with anti-CYP2E1 and Protein A Sepharose^™^ CL-4B (Cytiva, Tokyo, Japan) (which specifically supplements IgG) and immunoprecipitated. The precipitates were electrophoresed and Western blot was conducted. Then the protein was labeled with primary anti-CYP2E1 antibody (GeneTech, Tokyo, Japan) and secondary anti-rabbit IgG (horseradish peroxidase [HRP]-conjugated). The CYP2E1 protein was confirmed by visualization with chemiluminescent reagent compared to ones without the vector.

Serum anti-CYP2E1 IgG antibody levels were compared to confirm the titer of the synthesized CYP2E1 protein between using synthesized CYP2E1 and commercially available one (ATGen CYP2E1, Montevideo, Uruguay) (Supplementary Fig. 1) in randomly selected 15 serum samples as described below. The detected levels were significantly higher when using synthesized CYP2E1 protein than a commercial one, suggesting the higher purification of our synthesized CYP2E1 protein.

### Enzyme-linked immunosorbent assay for anti-CYP2E1 IgG antibody detection

We developed an enzyme-linked immunosorbent assay (ELISA), which was essentially similar to the method reported by Eliasson and Kenna (1996), to detect serum anti-CYP2E1 antibodies. The anti-CYP2E1 antibody levels in the sera of subjects in the TCE-HS, TCE-TC, and TCE-nonEC groups were determined using sandwich ELISA. A 96-well Immuno Plate (Thermo Fisher Scientific) was coated with CYP2E1 antibody (50 μL/well) (Gene Tex, Irvine, CA, USA), which was diluted in 1 × PBS at the ratio of 1:1000, by incubating the plate at 37 °C for 30 min. Then, 10% skim milk was added to the plate (200 μL/well), followed by incubation at room temperature for 30 min to block any nonspecific binding sites. After being washed with 1 × PBS three times, the plate was incubated with 2.7 μg/mL of CYP2E1 protein (50 μL/well) at room temperature for 30 min. After a wash step, the serum samples of subjects, diluted in Can Get Signal Immunoreaction Enhancer Solution 1 (TOYOBO, Tokyo, Japan) at the ratio of 1:800, was added to the plate, followed by incubation at room temperature for 30 min. Then, the blocking (5 min) and washing steps were performed, and the plate was incubated with anti-human IgG HRP-conjugated antibody (50 μL/well; Abcam, Cambridge, UK) at room temperature for 30 min, which was diluted in Can Get Signal Immunoreaction Enhancer Solution 2 (TOYOBO) at the ratio of 1:1000. After the blocking (5 min) and washing steps, *o*-phenylenediamine dihydrochloride (FUJIFILM, Tokyo, Japan) substrate solution, which was prepared according to the manufacturer's protocol, was added to the plate (50 μL/well), followed by incubation at room temperature for 30 min. Next, 3 N HCl was used (50 μL/well) to stop the reaction, and the absorbance at 490 nm was measured.

### CYP2E1 protein in sera

The level of human CYP2E1 protein in the sera of 68, 69, and 69 subjects in the TCE-HS, TCE-TC, and TCE-nonEC groups, respectively, was measured according to the manufacturer’s protocol (MyBioScource, San Diego, CA, USA).

### Statistical analysis

Categorical variables were compared using chi-squared test and Fisher’s exact test. Serum anti-CYP2E1 antibody (IgG) levels measured as absorbance were compared among the TCE-HS, TCE-TC, and TCE-nonEC groups using one-way (patient/control groups) or two-way (patient/control groups × sex) analysis of variance (ANOVA) followed by Bonferroni, or Steel–Dwass analysis in each of the two group pairs. In addition, effects of sex, drinking, and smoking habits, TCE exposure level (8 h TWA TCE concentration or urinary TCE metabolite concentrations) on serum anti-CYP2E1 antibody (IgG) and/or ALT levels were analyzed using multiple regression analysis.

We also investigated relationships between serum CYP2E1 antibody (IgG) levels and duration of TCE exposure, TCE TWA concentration, or urinary TCE metabolite (TCEOH and TCA) concentrations. TCE exposure durations in days were categorized into 0 (TCE-nonEC), 1–15, 16–30, 31–60, 61–90, 91–200, and over 200 days, based on the fact that the onset of TCE-HS is on average 1 month after the commencement of exposure (Huang et al. 2006; Kamijima et al. 2007). TCE TWA concentrations were stratified in consideration of the current Occupational Exposure Limit (OEL) (25 ppm) recommended by the Japan Society for Occupational Health (JSOH) (Japan Society for Occupational Health, 2020). The categorization of urinary TCEOH and TCA concentrations was made referring to OEL based on biological monitoring (OEL-B) of 100 mg/L and 50 mg/L, respectively, which JSOH also recommends. The criterion of significance was set at *p* < 0.05. Statistical analysis was performed using Stata 15.1 statistical software package (Stata Corp, College Station, TX, USA).

## Results

### Effects of sex, age, lifestyle, HLA-B*13:01 genotype, and ALT levels on anti-CYP2E1 IgG antibody levels

Profiles of the study subjects (Table 1) show that more number of men who matched to TCE-HS patients in terms of sex and age were recruited than women in the TCE-nonEC group. In contrast, more number of women were recruited in the TCE-TC group. No significant difference in age was observed among the three groups. In all groups, the percentages of smokers and drinkers were higher in men than in women. The order of their total percentages was TCE-nonEC > TCE-TC > TCE-HS patients.

**Table 1:** Profiles of the trichloroethylene hypersensitivity syndrome (TCE-HS) patients, TCE-tolerant controls (TCE-TC), and TCE-nonexposed controls (TCE-nonEC)

			TCE-HS patients	TCE-TC	TCE-nonEC
Age	Total *n*		24.1 ± 6.8 80	24.8 ± 6.6 186	25.1 ± 6.5 71
	Men *n* (%)		24.0 ± 7.2 51 (64)*	25.8 ± 6.3 87 (47)*	24.7 ± 6.6 47 (66)*
	Women *n* (%)		24.2 ± 6.0 29 (36)*	24.0 ± 6.7 99 (53)*	25.9 ± 6.4 24 (34)*
Smoker	Total *n* (%)		13 (16)	44 (24)	23 (32)
	Men *n* (%)		13 (25)^a^	44 (51)	22 (47)
	Women *n* (%)		0 (0)	0 (0)	1 (4)
Drinker	Total *n* (%)		11 (14)^a,b^	50 (27)^a^	38 (54)
	Men *n* (%)		11 (22)^a,b^	43 (50)^a^	37 (79)
	Women *n* (%)		0 (0)	7 (7)	1 (4)
Anti-CYP2E1 Absorbance	Total *n* (%)		0.412 ± 0.207^c,d^ 80 (100)	0.519 ± 0.204^c^ 186 (100)	0.244 ± 0.101^d^ 71 (100)
	Men *n* (%)		0.434 ± 0.227^c^ 51 (64)	0.475 ± 0.164^c,e^ 87 (47)	0.218 ± 0.089^d^ 47 (66)
	Women *n* (%)		0.375 ± 0.162^d^ 29 (36)	0.557 ± 0.228^c^ 99 (53)	0.293 ± 0.107^d^ 24 (34)
	Total *n* (%)	With HLA-B*13:01	0.361 ± 0.196 27 (100)	0.522 ± 0.172 19 (100)	ND
	Men *n*(%)	With HLA-B*13:01	0.387 ± 0.228 18 (67)	0.487 ± 0.106 10 (53)	ND
	Women *n* (%)	With HLA-B*13:01	0.308 ± 0.098 9 (33)	0.561 ± 0.225 9 (47)	ND
	Total *n*(%)	Without HLA-B*13:01	0.416 ± 0.174 11 (100)	0.558 ± 0.218 87 (100)	ND
	Men *n* (%)	Without HLA-B*13:01	0.418 ± 0.175 8 (73)	0.505 ± 0.175 40 (46)	ND
	Women *n*(%)	Without HLA-B*13:01	0.411 ± 0.209 3 (27)	0.505 ± 0.205 43 (54)	ND
Serum ALT (IU/L)	Total *n* (%)		752 ± 973 80 (100)	13.8 ± 16.0^c^ 181 (100)	20.3 ± 12.0 ^d^ 51 (100)
	Men *n* (%)		825 ± 1164 51 (64)	15.9 ± 10.8 ^c,e^ 83 (46)	25.3 ± 11.5 ^d,e^ 33 (65)
	Women *n* (%)		625 ± 471 29 (36)	12.1 ± 19.3 98 (54)	11.1 ± 6.1 18 (35)

Smokers included ex-smokers. Continuous variables are shown as mean ± standard deviation, and discontinuous variables are presented as frequency (%). Patients’ ALT levels, which were clearly higher than those of subjects in the TCE-TC and TCE-nonEC groups, were measured at the time of admission to the hospital; therefore, we did not conduct statistical tests. ALT levels of the TCE-TC and TCE-nonEC groups were log-transformed and statistically analyzed

*ND* not determined

*p < 0.05 by Chi-squared test

^a^Significantly different from the TCE-nonEC group using the Bonferroni–Holm method after the chi-square test (*p* < 0.05)

^b^Significanty different from the TCE-TC group using the Bonferroni–Holm method after the chi-square test (*p* < 0.05)

^c^Significantly different from the TCE-nonEC group using the Bonferroni method after one-way (patient/control groups) or two-way (patient/control groups × sex) analysis of variance (*p* < 0.05)

^d^Significantly different from the TCE-TC group using the Bonferroni method after one-way (patient/control groups) or two-way (patient/control groups × sex) analysis of variance (*p* < 0.05)

^e^Significantly different from women in the same group using the Bonferroni method after two-way (patient/control groups × sex) analysis of variance (*p* < 0.05)

**Fig. 1:**
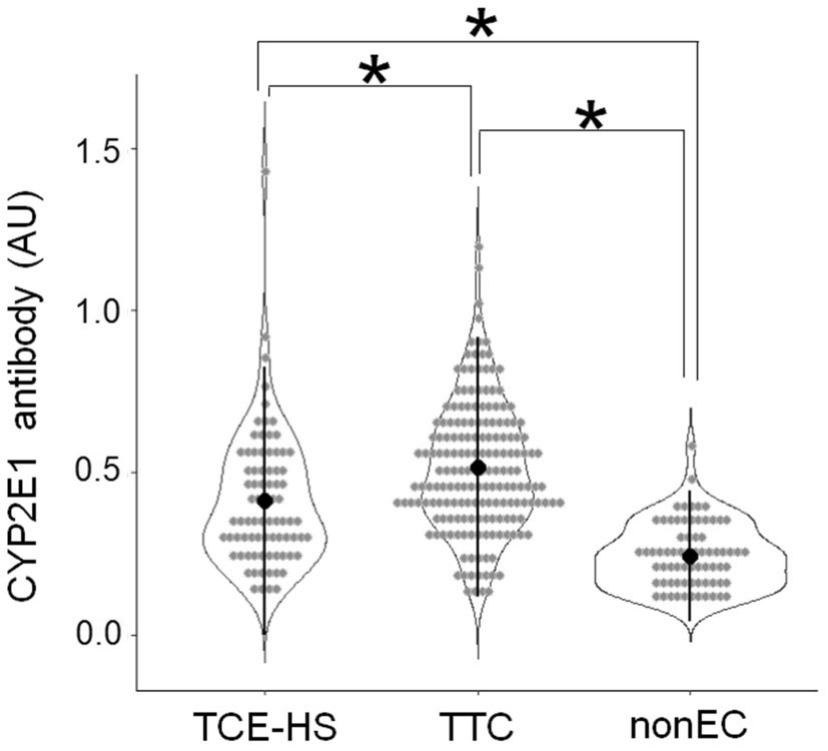
Violin plot analysis comparing anti-CYP2E1 antibody (IgG) levels among the trichloroethylene hypersensitivity syndrome (TCE-HS), TCE-tolerant control (TTC), and TCE-nonexposed control (TCE-nonEC) groups. The vertical axis represents the absorbance at 490 nm. Light dots indicate individual measured data, and the dark dot and vertical line in each plot denote the mean and standard deviation, respectively. *AU* absorbance unit. *p < 0.05

The individual anti-CYP2E1 antibody (IgG) levels in the TCE-HS, TCE-TC, and TCE-nonEC groups were normally distributed (Fig. 1), and two-way ANOVA showed a significant difference in anti-CYP2E1 IgG antibody levels among the three groups, i.e., TCE-TC > TCE-HS patients > TCE-nonEC (Table 1 and Fig. 1). There was no gender difference in the TCE-HS and TCE-nonEC groups, but a gender difference was observed in the TCE-TC group, with a higher levels of women than men. In other words, the induction of serum anti-CYP2E1 antibodies (IgG) by TCE exposure was stronger in women. Anti-CYP2E1 antibody levels were then compared between HLA-B*13:01 carriers and noncarriers, but no significant difference was observed in either patients or TCE-TC.

The serum ALT levels of all TCE-HS patients were extremely high and deviated from the normal range. On the other hand, of the serum ALT levels of 181 TCE-TC and 51 TCE-nonEC, the values of 7 and 4 people, respectively, showed abnormal liver function. When the ALT values were log-transformed and compared between the groups, the values of TCE-nonEC were higher than those of TCE-TC. Two-way ANOVA showed that the interaction was significant. Regarding between-group differences, a significant difference was observed between the TCE-TC and TCE-nonEC groups for men only, and sex differences were observed in both groups.

### Multiple regression analysis for anti-CYP2E1 IgG antibody and ALT values

Multiple regression analysis that was conducted in TCE-TC and TCE-nonEC for anti-CYP2E1 antibody (IgG) levels as the objective variable and TCE exposure status, sex, age, smoking habit, drinking habit, and ALT level as explanatory variables revealed that TCE exposure and women had a positive effect on anti-CYP2E1 autoantibody (IgG) induction (Table 2). When the number of days of TCE exposure, TWA TCE concentrations, urinary TCA, or TCEOH was added to these explanatory variables, the effects of factors affecting CYP2E1 antibody (IgG) disappeared completely (data not shown).

**Table 2:** Multiple regression analysis of anti-CYP2E1 antibody levels in TCE-tolerant controls (TCE-TC) and TCE-nonexposed controls (TCE-nonEC)

Explanation factor		Total *n* = 231 *r*^*2*^ = 0.2963	Men *n* = 115 *r*^*2*^ = 0.3870	Women *n* = 116 *r*^*2*^ = 0.1626
TCE exposure status 0, TCE-nonEC 1,TCE-TC	*β* (95% CI) *p*	0.496 (0.379, 0.614) < 0.001	0.630 (0.463, 0.797) < 0.001	0.414 (0.235, 0.594) < 0.001
Age	*β* (95% CI) *p*	0.045 (− 0.069, 0.158) 0.440	0.028 (− 0.123, 0.179) 0.712	0.056 (− 0.126, 0.237) 0.545
Sex 0, Men; 1, Women	*β* (95% CI) *p*	0.159 (0.001, 0.318) 0.049		
Smoking habit 0, No; 1, Yes	β (95% CI) *p*	− 0.030 (− 0.162, 0.116) 0.659	− 0.050 (− 0.199, 0.100) 0.513	0.031 (− 0.147, 0.208) 0.732
Drinking habit 0, No; 1, Yes	β (95% CI) *p*	− 0.005 (− 0.128, 0.138) 0.940	0.025 (− 0.129, 0.178) 0.751	− 0.018 (− 0.192, 0.156) 0.837
log_10_ ALT levels (IU/L)	β (95% CI) *p*	0.009 (− 0.122, 0.140) 0.895	0.008 (− 0.158, 0.173) 0.927	0.006 (− 0.174, 0.187) 0.946

ALT values were log-transformed

*β* partial correlation coefficient, *95% CI* 95% confidence interval

The effects of age, sex, alcohol and smoking habits, and TCE exposure status mentioned above on ALT levels were analyzed by multiple regression analysis (Table 3). TCE exposure and women (men 0, women 1) negatively affected ALT, while age had positive effects on the levels. However, drinking and smoking habits did not affect the levels. Therefore, TCE exposure alone did not increase serum ALT levels.

**Table 3:** Multiple regression analysis of ALT levels in TCE-tolerant controls (TCE-TC) and TCE-nonexposed controls (TCE-nonEC)

Explanation factor		Total *n* = 244 *r*^*2*^ = 0.2817	Men *n* = 123 *r*^*2*^ = 0.1676	Women *n* = 121 *r*^*2*^ = 0.0813
TCE exposure status 0, TCE-nonEC; 1, TCE-TC	β (95% CI) *p*	− 0.233 (− 0.347, − 0.119) < 0.001	− 0.397 (− 0.572, − 0.221) < 0.001	− 0.057 (− 0.244, 0.130) 0.547
Age	β (95% CI) *p*	0.169 (0.057, 0.280) 0.003	0.150 (− 0.020, 0.321) 0.083	0.249 (0.066, 0.432) 0.008
Sex 0, Men; 1, Women	β (95% CI) *p*	− 0.418 (− 0.568, − 0.268) < 0.001		
Smoking habit 0, No; 1, Yes	β (95% CI) *p*	− 0.062 (− 0.195, 0.071) 0.358	− 0.066 (− 0.237, 0.104) 0.443	− 0.011 (− 0.196, 0.173) 0.905
Drinking habit 0, No; 1, Yes	β (95% CI) *p*	− 0.009 (− 0.125, 0.143) 0.895	0.035 (− 0.141, 0.211) 0.693	− 0.102 (− 0.282, 0.079) 0.266

ALT values were log-transformed

*β* partial correlation coefficient, *95% CI* confidence interval

### Serum CYP2E1 protein

Table 4 presents serum CYP2E protein levels in the TCE-HS, TCE-TC, and TCE-nonEC groups. No significant difference in the serum CYP2E1 protein levels was observed between the control groups. Interestingly, the serum CYP2E protein levels in the TCE-HS group were significantly higher than those in the two control groups. No difference in the serum CYP2E1 protein levels was observed in any group in terms of sex (data not shown).

**Table 4:** CYP2E1 protein levels in the sera of TCE-HS patients, TCE-tolerant controls (TCE-TC), and TCE-nonexposed controls (TCE-nonEC)

		TCE-HS patients	TCE-TC	TCE-nonEC
CYP2E1 levels (ng/ml)	Total *n*	162.4 ± 646.3^a^ 68	7.4 ± 10.0 69	20.0 ± 74.5 69

Variables are shown as mean ± standard deviation

^a^Steel–Dwass test results show significant difference (*p* < 0.05) between patients and other groups

### Effects of TCE exposure duration on serum anti-CYP2E1 antibody (IgG) levels

**Fig. 2:**
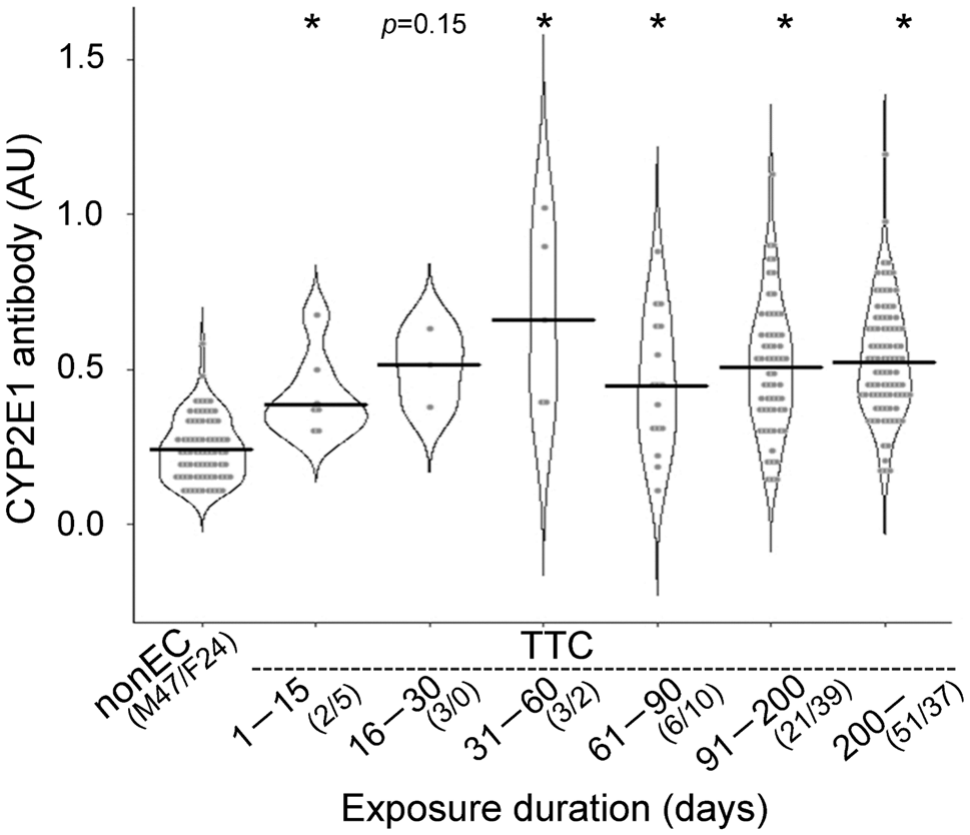
Violin plot analysis comparing anti-CYP2E1 antibody (IgG) levels according to TCE exposure durations in control workers. The vertical axis represents the absorbance at 490 nm. Dots and horizontal bars indicate individual measured data and the median of each group, respectively. The numbers in the parentheses are men (M)/women (F). *nonEC* TCE-nonexposed control, *TTC* TCE-tolerant control, *AU* absorbance unit. *p < 0.05

Figure 2 shows the distributions and medians of serum anti-CYP2E1 antibody (IgG) levels of the TCE-nonEC (*n* = 71) and TCE-TC groups (*n* = 179 with exposure duration data available) in each TCE exposure duration category. Of the recruited workers, only seven were engaged in TCE-exposed jobs for 15 days or fewer, accounting for 3.9% of the total, and their serum anti-CYP2E1 antibody (IgG) levels were significantly higher than those of subjects in the TCE-nonEC group. The median serum anti-CYP2E1 antibody (IgG) level of workers exposed to TCE for 16–30 days (*n* = 3) tended to be higher, but the difference was not statistically significant (Fig. 2). Meanwhile, five workers (2.8% of the total) were engaged in the TCE-exposed jobs for 31–60 days, and TCE exposure significantly increased the serum anti-CYP2E1 antibody (IgG) levels in these subjects compared with TCE-nonEC. Interestingly, the serum anti-CYP2E1 antibody (IgG) levels in the ≥ 61-day exposure group (164 workers, 91.6%), which were also significantly higher than those in the TCE-nonEC group, did not continue to increase compared with the 31–60-day exposure group.

### Effects of TCE TWA concentrations on anti-CYP2E1 antibody (IgG) levels

**Fig. 3:**
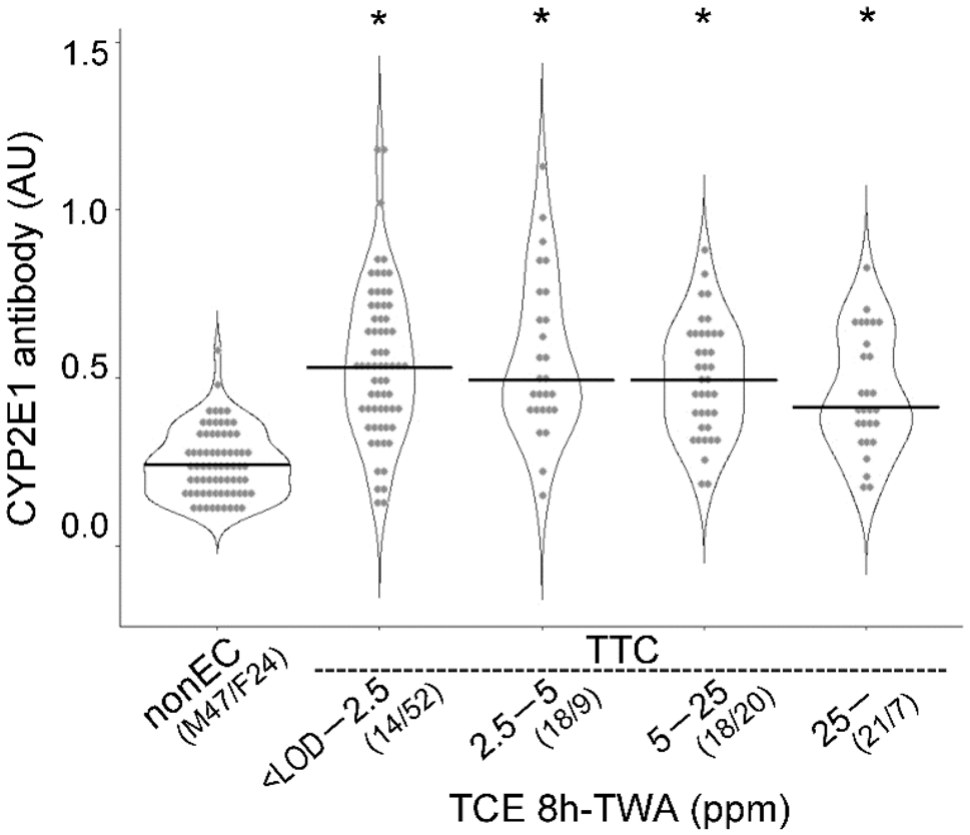
Violin plot analysis comparing anti-CYP2E1 antibody (IgG) levels according to 8 h-TWA TCE concentration categories in control workers. See the legend in Fig. 2 for more details about the graph. *nonEC* TCE-nonexposed control, *TTC* TCE-tolerant control. *p < 0.05

We measured airborne TWA TCE concentrations of 159 workers out of 186 who engaged in TCE-exposed jobs. Figure 3 shows the distributions and medians of serum anti-CYP2E1 antibody (IgG) levels according to each TCE concentration range. Twenty-eight (17.6%) workers whose TWA concentrations exceeded the TCE OEL of 25 ppm recommended by the JSOH. Surprisingly, TCE increased serum anti-CYP2E1 antibody (IgG) levels at very low concentrations from LOD to 2.5 ppm (66 workers, 41.5%), which was one-tenth of the current OEL, to the same extent as at higher concentration levels. Obviously, individual differences in antibody levels in the two lowest exposure groups (< LOD – 2.5 ppm and 2.5 < – 5 ppm) were very large.

### Relationship between urinary TCE oxidative metabolites and serum anti-CYP2E1 antibody (IgG) levels

**Fig. 4:**
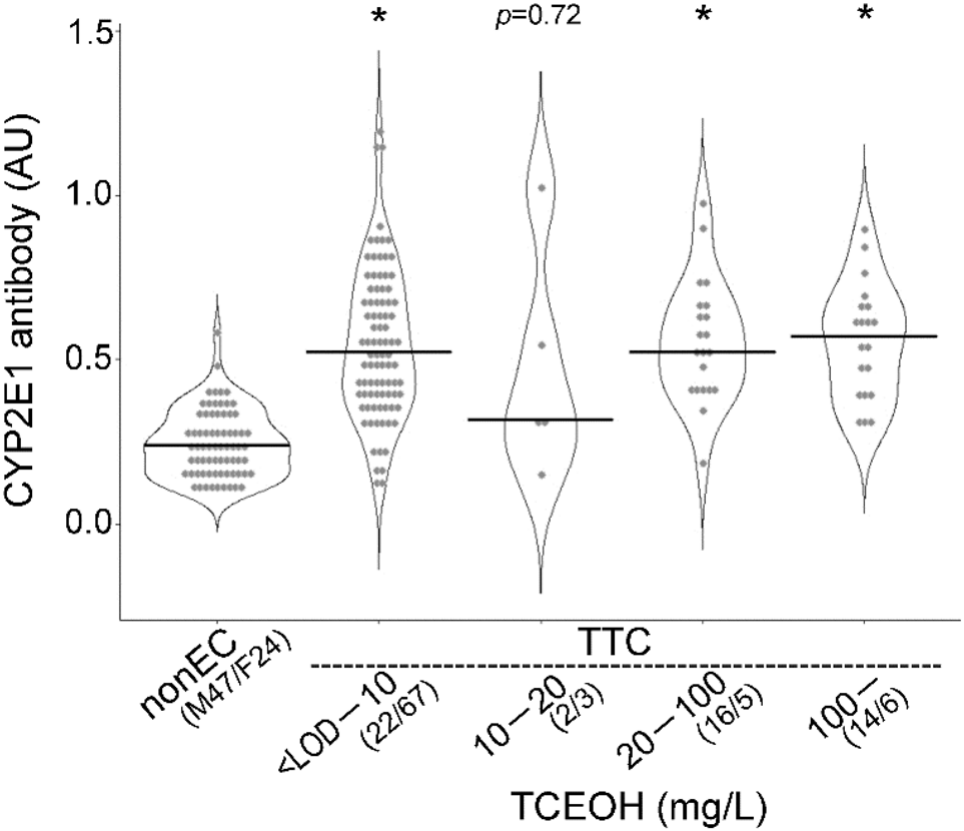
Violin plot analysis comparing anti-CYP2E1 antibody (IgG) levels according to urinary TCEOH levels in control workers. See the legend in Fig. 2 for more details about the graph. *nonEC* TCE-nonexposed control, *TTC* TCE-tolerant control. *p < 0.05

Urinary TCE oxidative metabolites TCEOH and TCA of 135 TCE-exposed workers were measured by GC–MS, and serum anti-CYP2E1 antibody (IgG) levels were compared among the different concentration groups. Based on OEL-B of 100 mg/L for TCEOH recommended by the JSOH, the workers were divided into four groups: <LOD to 10 mg/L, > 10 mg/L to 20 mg/L, > 20 mg/L to 100 mg/L, and > 100 mg/L (Fig. 4). In 135 workers, urinary TCEOH level of 20 (14.8%) workers exceeded the OEL-B, which showed a similar frequency to those who exceeded the airborne OEL of 25 ppm (28 persons, 17.6%). Compared with TCE-nonEC, serum anti-CYP2E1 antibody (IgG) levels were significantly increased in the group with the lowest TCEOH concentration level, showing approximately the same level of increase as in the higher concentration groups, though the increase in the second-lowest group was not significant. It is noted that the distribution of anti-CYP2E1 antibody (IgG) in the lowest group of urinary TCEOH was also very large like that in the lowest TCE TWA concentration group.

**Fig. 5:**
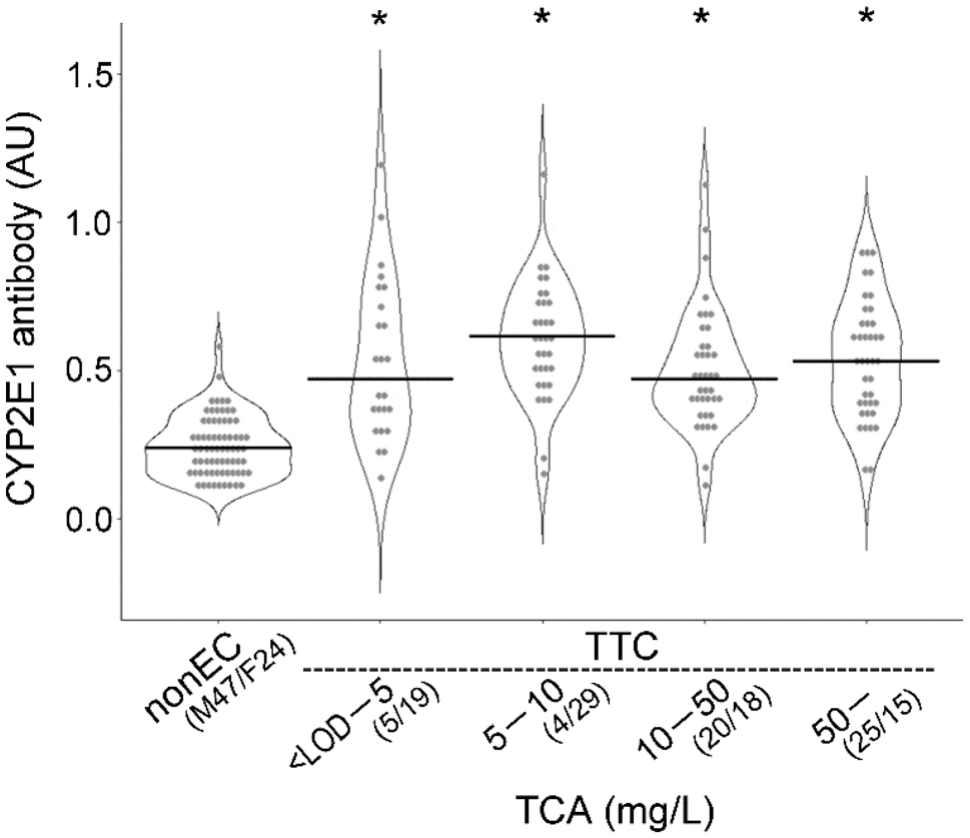
Violin plot analysis comparing anti-CYP2E1 antibody (IgG) levels according to urinary TCA levels in control workers. See the legend in Fig. 2 for more details about the graph. *nonEC* TCE-nonexposed control, *TTC* TCE-tolerant control. *p < 0.05

Regarding urinary TCA, we divided TCE-TC into four sub-groups by referring to OEL-B in Japan: <LOD to 5 mg/L (1/10 of OEL-B), > 5 to 10 mg/L (1/5 of OEL-B), > 10 to 50 mg/L (OEL-B), and > 50 mg/L (Fig. 5). Serum CYP2E1 antibody (IgG) levels significantly increased in the lowest group of urinary TCA; however, when the TCA concentration increased, it did not increase further. The distribution of TCE workers in each subgroup was similar (24.4–29.6%) except for the lowest group (17.8%). Of note, the number of workers (40 persons, 29.6%) in the highest concentration group (> 50 mg/L) was about twice the number of workers with TCEOH of more than 100 mg/L and of those with TWA exposure concentrations exceeding OEL 25 ppm. Given the long biological half-life of TCA of 83.7 h in this patient population (Nakajima et al. 2018), the measured urinary TCA levels may have reflected higher TCE exposure concentrations of the preceding days before urine collection.

## Discussion

It is well known that TCE exposure can cause immunological diseases (Cooper et al. 2009), and TCE-HS is one of them. However, the pathogenesis of this condition has not been clarified except for the involvement of HLA-B*13:01 polymorphism (Li et al. 2007; Wang et al. 2020) and HHV6 reactivation (Huang et al. 2006; Kamijima et al. 2013; Watanabe et al. 2010). The present study showed that TCE induced serum anti-CYP2E1 autoantibody (IgG) and suggested that the autoantibody played an essential role in inducing hepatotoxicity in patients with TCE-HS, representing a major clinical manifestation other than skin damage.

In the TCE-TC group, the autoantibody levels tended to be related to the duration of TCE exposure, which might explain why TCE-HS occurred after repeated exposure (Huang et al. 2006; Kamijima et al. 2007). Although TCE exposure is essential for this induction, we could not clarify the lowest observed adverse effect concentration in terms of external exposure concentrations (TWA) or internal exposure, i.e., the urinary metabolite concentrations. Serum anti-CYP2E1 antibodies are reportedly elevated by alcohol consumption (Lytton et al. 1999), but neither alcohol consumption nor smoking habits affected its levels in our study. Women had higher anti-CYP2E1 autoantibody levels than men, but the mechanism behind this finding is unknown.

The autoantibodies were also detected in the sera of patients diagnosed with TCE-HS. However, their levels were lower than those of TCE-TC, while significantly higher than those of TCE-nonEC. These data might be considered the result of increased binding of the autoantibodies to the higher levels of antigens (i.e., CYP2E1) in the blood, because our developed method for measuring anti-CYP2E1 IgG antibodies could not theoretically measure antibodies bound to CYP2E1 protein. This interpretation is supported by the fact that the MyBioSource kit could measure CYP2E1 protein levels but could not distinguish free CYP2E1 protein from that bound to immunocomplexes. The levels of CYP2E1 protein were elevated only in patients with TCE-HS, whereas its levels were much lower in the TCE-TC and TCE-nonEC groups without differences between these groups. The CYP2E1 protein in patients with TCE-HS was likely bound to the autoantibodies, and the autoantibody levels must have been higher than values reported in this study and those measured in the TCE-TC group immediately before the onset of HS. Therefore, it is not surprising that ALT levels were not elevated in the TCE-TC group following TCE exposure. In patients with TCE-HS, serum CYP3A4 protein levels were not elevated (data not shown). This finding is similar to that in patients with halothane hepatitis, and it might indicate that CYP3A4 was not involved in the development of TCE-HS.

It has been studied in detail that exposure to some chemicals induces the production of serum anti-CYP2E1 antibodies involved in the pathogenesis of hepatitis, especially halothane hepatitis (Sutti et al. 2014). Walton et al. (1976) reported that repeated exposure to halothane within 4 weeks might cause immune hepatitis. Patients with halothane hepatitis had higher levels of serum anti-liver-kidney-microsomal antibodies, thyroid antibodies, and autoimmune complement than those who were tolerant to halothane hepatitis. This suggests that halothane hepatitis may be (type II) autoimmune hepatitis. Eliasson and Kenna (1996) reported that anti-CYP2E1 antibody levels in sera from patients with halothane hepatitis were higher than in halothane-tolerant controls, healthy controls, patients with primary biliary cirrhosis, and other liver diseases, directly proving evidence that halothane hepatitis is a CYP2E1-mediated autoimmune disease. Anti-CYP2E1 antibody is also induced by HCFC-123 (White and De Matteis 2001) and ethanol (Lytton et al. 1999) as well. All these chemicals have in common that CYP2E1 is the metabolizing enzyme. This isozyme metabolizes halothane and HCFC-123 to the active metabolite CF_3_COCl that binds to liver proteins and forms trifluoroacetylated protein in the liver, which becomes the antigen (Hoet et al. 1997; Kenna and Neuberger 1995). Eliasson and Kenna (1996) reported that this trifluoroacetylated protein complex was detected in the sera of 70% of halothane hepatitis patients but that CYP3A did not create such a complex. Four of six HCFC-123-affected workers had positive CYP2E1 autoantibodies trifluoroacetyl-adducted CYP2E1 in their sera (Hoet et al. 1997). Similar to halothane and HCFC-123, TCE is metabolically activated by CYP2E1. The active metabolite(s) may not be chloral hydrate (CH), but it might be an intermediate produced between TCE and CH (Nakajima et al. 1988). Concerning the fact that TCE causes immune diseases such as Lupus and scleroderma (Byers et al. 1988; Flindt-Hansen and Isager 1987; Hansen and Isager 1988) and trichloroacetaldehyde (TCAH) hydrate (similar to CH) is the active metabolite (Blossom and Gilbert 2006; Gilbert et al. 2006), its effect on the immune system was investigated using autoimmune-prone MRL +/+ mice. As a result, skin alopecia and inflammation, increased serum IgM and IgG2, and increased interferon-gamma in CD4^+^ T cells were reported in i*n vitro* experiments (Blossom et al. 2007). However, whether trichloroacetaldehyde hydrate induces hepatitis has not been investigated. Later, Gilbert et al. (2009) observed lymphocytic infiltration in the liver of MRL +/+ mice after prolonging the TCE exposure period to 26 weeks, but there is no report regarding which metabolites are involved in liver damage. More recently, Li et al. (2019) demonstrated in vitro that TCAH, but not TCA or TCEOH, activated CD4^+^ T cells, which are involved in all aspects of autoimmune disease. Thus, the common mechanism appears to be the breakdown of self-tolerance to CYP2E1 and autoantibody production in response to active metabolites at the onset of immune hepatitis.

Anti-CYP2E1 autoantibodies are considered a biomarker of drug-induced liver injury (McCarthy et al. 2018). CYP2E1 is mainly localized in the endoplasmic reticulum but is also expressed in mitochondria and the outer layer of the plasma membrane of liver cells (Lu and Cederbaum 2008; Neve and Ingelman-Sundberg 2000). CYP2E1 in the plasma membrane is also active: the epitope of the steric hindrance locus is located in the G-helix and J–L helix regions of the outer membrane of the plasma membrane and binds easily to antibodies (Sutti et al. 2014). Halothane exhibits antibody-mediated cytotoxicity, but it is also thought to respond to T-cell-derived CYP2E1 (Sutti et al. 2014). TCE may induce antibody-dependent cellular cytotoxicity similarly as halothane. However, Sutti et al. (2014) concluded that more studies are needed to characterize the impact of antibodies such as anti-CYP2E1 in type II autoimmune hepatitis.

**Fig. 6:**
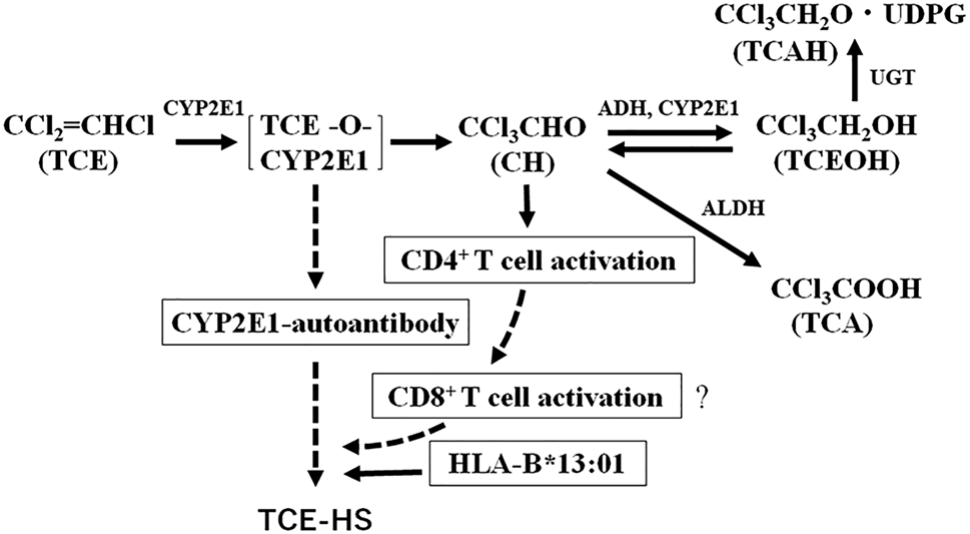
Oxidative metabolic pathway of TCE (IARC Working Group on the Evaluation of Carcinogenic Risks to Humans 2014) with slight modifications and the hypothesis of TCE-HS pathogenesis: involvement of CD4^+^ T cell activation (Li et al. 2019) and the subsequent activation of CD8^+^ T cells, anti-CYP2E1 autoantibody generation, and HLA-B*13:01 genetic polymorphism. Thick arrows indicate established paths, and dashed arrows indicate hypothetical paths. *TCE* trichloroethylene, *TCAH* trichloroacetaldehyde, *TCA* trichloroacetic acid, *TCEOH* trichloroethanol, *ADH* alcohol dehydrogenase, *ALDH* aldehyde dehydrogenase, *UGT* UDP-glucronosyltransferase, *HS* hypersensitivity syndrome

Therefore, we would like to discuss anti-CYP2E1 autoantibody generation and TCE-HS pathogenesis. As mentioned previously, circulating immune complexes were likely generated in the sera of patients with TCE-HS, and their preonset anti-CYP2E1 antibody levels must have been higher than the reported values and higher than those in the TCE-TC and TCE-nonEC groups. Because serum CYP2E1 protein levels were not different between the TCE-TC and TCE-nonEC groups, the immune complex levels may have been similar in these groups. It was also reported that TCAH activates CD4^+^ T cells (Li et al, 2019), which in turn are expected to activate CD8^+^ T cells. These three factors, i.e., circulating immune complexes, autoantibodies, and activated CD4^+^ T cells, may be involved in triggering TCE-HS (Fig. 6). However, we must remember the involvement of the susceptibility factor HLA-B*13:01 in the pathogenesis of TCE-HS (Li et al. 2007). As reported in our previous study (Wang et al. 2020), there were more HLA-B*13:01 carriers in the TCE-HS group (69.2%) than those in the TCE-TC (18.7%), which was also confirmed in the present study (29/39, 74.0% and 119/106, 7.9%, respectively). A comparison of CYP2E1 antibody levels between HLA-B*13:01 carriers and noncarriers revealed no difference in the TCE-TC or TCE-HS group, suggesting that the genotype may not be involved in the generation of this autoantibody. Instead, HLA-B*13:01 polymorphism may be involved in T-cell activation rather than autoantibody production. Zhang et al. (2013) analyzed 57 peptides that bind HLA-B*13:01. These peptides include a number of MHC class I antigens. However, the antigens involved in the pathogenesis of TCE-HS remain to be clarified.

Because CYP2E1 is also expressed in the skin (Neis et al. 2010; Oesch et al. 2018; Saeki et al. 2002; Yengi et al. 2003), it remains to be determined whether autoantibodies cause systemic dermatitis, which is the characteristic of TCE-HS (Huang et al. 2006; Kamijima et al. 2013), by binding to antigens in the skin or whether this event is attributable to the involvement of the circulating immune complexes, which might lead to keratinocyte apoptosis. In all patients with TCE-HS, HHV6, which was latent, was reactivated (Kamijima et al. 2013). In addition, we previously reported that the nonspecific IgE levels were slightly but significantly higher (*p* = 0.001) in the TCE-HS group (212.5 ± 88.0 IU/mL, *n* = 30) than those in the TCE-TC group (137.6 ± 75.6 IU/mL, *n* = 30) (Huang et al. 2006). The involvement of these events also needs to be considered further, but there are not many reports of concomitant skin damage with halothane hepatitis and none with HCFC-123 hepatitis, and information about HHV6 reactivation in this autoimmune hepatitis has not been reported.

There are a few limitations in the present study. The average duration of TCE exposure before disease onset is approximately a month (Huang et al. 2006; Kamijima et al. 2007, 2013). However, only eight subjects met this duration in this study. In addition, our study was a case–control study and not a prospective cohort study, which made it impossible to clarify the kinetics of anti-CYP2E1 antibodies from the beginning of TCE exposure. In addition, the level of autoantibodies required to trigger the onset of TCE-HS was not determined. Because there was an upward trend in anti-CYP2E1 antibodies from the beginning of exposure, a further follow-up study from the beginning of TCE-exposed work is warranted. Finally, because this study could not directly measure the levels of immune complexes circulating in the blood, it would be desirable to develop a method to measure them.

## Conclusion

Repeated exposure to TCE may increase serum anti-CYP2E1 autoantibodies, which might result in the onset of TCE-HS. We could not estimate the lowest limit of TCE exposure concentration that induces autoantibody production. The previously reported susceptibility gene HLA-B*13:01 is not involved in the induction of autoantibodies but may be involved in the subsequent autoimmune process and contribute to TCE-HS development.

## Supplementary Information

Below is the link to the electronic supplementary material.

Supplementary file1 (DOCX 17 KB)

## Data Availability

Part of HLA-B*13:01 genotypes reported in this study were taken from Wang et al., Environmental Research 2020; 191:109972, https://doi.org/10.1016/j.envres.2020.109972.

## Abbreviations

TCE: Trichloroethylene
HS: Hypersensitivity syndrome
HHV6: Human herpesvirus 6
HLA: Human leukocyte antigen
CYP: Cytochrome P450
TCE-TC: TCE-tolerant controls
TCE-nonEC: TCE-nonexposed controls
ALT: Alanine aminotransferase
GC–MS: Gas chromatograph-mass spectrometer
LOD: Limit of detection
TCA: Trichloroacetic acid
TCEOH: Trichloroethanol
ELISA: Enzyme-linked immunosorbent assay
OEL: Occupational exposure limit
JSOH: Japan Society for Occupational Health
OEL-B: OEL based on biological monitoring
TCAH: Trichloroacetaldehyde
