# Supplementary material for: Increased serum anti-CYP2E1 IgG autoantibody levels may be involved in the pathogenesis of occupational trichloroethylene hypersensitivity syndrome: a case–control study
Source: Arch Toxicol. 2022 Jun 28;96(10):2785–97. doi: 10.1007/s00204-022-03326-x (PMC9352743; doi:10.1007/s00204-022-03326-x)
Supplement: Supplementary file 1 — Supplementary file1 (DOCX 17 KB) [file 204_2022_3326_MOESM1_ESM.docx]

Supplementary Fig.1.

**Increase in serum CYP2E1 autoantibody may be involved in pathogenesis of occupational trichloroethylene hypersensitivity syndrome: A case-control study**

***^*^ Tamie Nakajima,^1,*^ Hailan Wang,^2,*^ Yuan Yuan,^1^ Yuki Ito,^3^ Hisao Naito,^4^ Yoshiyuki Kawamoto,^5^ Kiyoshi Sakai,^3^ Na Zhao,^2^ Hongling Li,^2^ Xinxiang Qiu,^6^ Lihua Xia,^6^ Jiabin Chen,^6^ Qifeng Wu,^6^ Laiyu Li,^2^ Hanlin Huang,^7^ Yukie Yanagiba,^8^ Hiroshi Yatsuya,^9^ and Michihiro Kamijima^3^***

Address correspondence to Tamie Nakajima, Research Institute of Life and Health Sciences, Chubu University, Kasugai, 487-8501 Japan. E-mail: [tnasu23@med.nagoya-u.ac.jp](mailto:tnasu23@med.nagoya-u.ac.jp)
